# Antifungal and Aflatoxin-Reducing Activity of β-Glucan Isolated from *Pichia norvegensis* Grown on Tofu Wastewater

**DOI:** 10.3390/foods10112619

**Published:** 2021-10-28

**Authors:** Gemilang Lara Utama, Mahardhika Puspa Arum Suraloka, Tita Rialita, Roostita Lobo Balia

**Affiliations:** 1 Master Program of Agro-Industrial Technology, Faculty of Agro-Industrial Technology, University of Padjadjaran, Jatinangor 45363, Indonesia; kha_suraloka@yahoo.com (M.P.A.S.); tita.rialita@unpad.ac.id (T.R.); 2 Centre for Environment and Sustainability Science, University of Padjadjaran, Bandung 40134, Indonesia; 3 Veterinary Study Program, Faculty of Medicine, University of Padjadjaran, Jatinangor 45363, Indonesia

**Keywords:** *Pichia*, β-glucan, antifungal, aflatoxin reducer

## Abstract

Yeast can be isolated from tofu wastewater and the cell wall in the form of β-glucan can act as a natural decontaminant agent. This study aimed to isolate and characterize native yeast from tofu wastewater, which can be extracted to obtain β-glucan and then identify the yeast and its β-glucan activity regarding antifungal ability against *Aspergillus flavus* and aflatoxin-reducing activity towards aflatoxin B1 (AFB1) and B2 (AFB2). Tofu wastewater native yeast was molecularly identified, and the growth observed based on optical density for 96 h and the pH also measured. β-glucan was extracted from native yeast cell walls with the acid-base method and then the inhibition activity towards *A. flavus* was tested using the well diffusion method and microscopic observation. AFB1 and AFB2 reduction were identified using HPLC LC-MS/MS. The results showed that the native yeast isolated was *Pichia norvegensis* with a β-glucan yield of 6.59%. *Pichia norvegensis* and its β-glucan showed an inhibition zone against *Aspergillus flavus* of 11.33 ± 4.93 and 7.33 ± 3.51 mm, respectively. Total aflatoxin-reducing activity was also shown by *Pichia norvegensis* of 26.85 ± 2.87%, and β-glucan of 27.30 ± 1.49%, while AFB1- and AFB2-reducing activity by *Pichia norvegensis* was 36.97 ± 3.07% and 27.13 ± 1.69%, and β-glucan was 27.13 ± 1.69% and 32.59 ± 4.20%, respectively.

## 1. Introduction

Tofu wastewater from the tofu industry is generated in large amounts; thus, if it is not utilized and thrown away, it will have a bad impact on the environment. Tofu wastewater contains very high organic substances with low acidity at pH 5–6 [1]. The largest component of tofu wastewater is protein (*N*-total) of 226.06 to 434.78 mg/L, while the gases found in tofu wastewater disposal are nitrogen gas (N_2_), oxygen (O_2_), hydrogen sulfide (H_2_S), ammonia (NH_3_), carbon dioxide (CO_2_), and methane (CH_4_) [2]. These gases come from the decomposition of organic materials contained in the wastewater and in general the hydrogen ion concentration of the tofu wastewater tends to be acidic. The temperature of tofu wastewater in general is 40–46 °C and if it is thrown away into the environment, the increased temperature in the aquatic environment will affect biological life, the solubility of oxygen and other gases, water density, viscosity, and surface tension [3]. Therefore, one way to reduce these problems is to utilize tofu wastewater.

Tofu wastewater has been known to contain organic compounds that can be used as substrates for the growth of microorganisms. Tofu wastewater can be applied as a cheap medium and raw material for growing microbes as well as providing new opportunities to produce biofunctional compounds [4]. Maryana et al. [5] utilized tofu wastewater as a growth medium for *Rhizopus oryzae* to produce single-cell proteins. Meanwhile, Utami and Suprihadi [6] used tofu wastewater as a growth medium for *Aspergillus flavus* DUCC-K225 to produce protease enzymes. The optimum time for *S. cerevisiae* growth in tofu wastewater was 48 h with a number of colonies and cell dry weight of 50 × 10^7^ CFU/mL and 0.049 g/mL, respectively [7].

This research utilized the tofu wastewater as a growth medium for yeast to produce β-glucan. The yeast grown is native yeast isolated from the tofu wastewater, so it is necessary to isolate and identify the yeast from the tofu wastewater. Native yeast from tofu wastewater that has been identified molecularly is then grown and extracted to produce β-glucan.

Although scarce research has been found regarding the identification of yeast types isolated from tofu wastewater, Xu et al. [8] stated that 16 yeasts were isolated from naturally fermented acid slurry of soy whey; however, no identification of the yeast type was made. Meanwhile, according to Li et al. [9], yeast isolated and identified from tofu whey (acidic tofu whey) is *Pichia amenthionina*. Tofu wastewater is produced from the residue of soybean cooking. Several references are used to examine and identify yeasts from soybean-based products, including soybean paste and tempeh [10]. Yeasts found in soybean paste include *Pichia guilliermondii* and *Candida fermentati* while yeast isolated from fermented tempeh include *Candida famata*, *Candida pelliculosa*, and *Candida lusianiae* [10,11].

Based on some studies, it is suspected that yeasts isolated from tofu wastewater include *Pichia* sp. or *Candida* sp., which have cell walls. According to Narusaka et al. [12], the yeast cell wall contains polysaccharides, such as polymers of glucose (β-glucan) and polymers of mannose (mannoprotein). The cell wall of yeast has a weight of 30% of the total number of cells and the cell wall content is mostly composed of mannoproteins and β-glucans with (1,3) bonds followed by β-glucan bonds (1,6) and chitin [13]. The content of manoprotein in the dry weight of the cell wall reaches 35–40%, chitin is about 1–2%, β-1.6 glucan is 5–10%, and more than half of the cell wall (50–55%) is composed of β-1.3 glucans [14]. The inner layer of the *Candida albicans* cell wall consists of β-glucan and chitin. β-glucan is the main component, which covers about 50–60% of the weight of the cell wall while chitin covers 1–10% of the weight of the cell wall [15].

*Candida* sp. produces β-glucan from its cell wall structure as well as *Pichia* sp., but each yeast will produce different characteristics of β-glucan. The difference in microorganisms will determine the characteristics of the β-glucan produced due to differences in their respective sources [16]. The biological activity of β-glucan is determined by its molecular weight and conformation, so the β-glucan obtained from each microorganism, even though it has the same basic structure, has different biological activity properties [17].

Yeast and β-glucan extracted from yeast cell walls are thought to have potential as antifungals. *Pichia membranifaciens* showed antifungal activity against post-harvest disease of grapes of *Botrytis cinerea* [18]. These yeasts can secrete exo- and endo-L-1,3-glucanase can thus inhibit the growth of molds [19]. *Pichia pastoris* has antifungal activity due to the hydrolytic action of chitin, which can interfere with and inhibit the growth of fungi [20]. Meanwhile Narusaka et al. [12] stated that yeast cell walls extracted from *S. pastorianus* containing ±40% mannoprotein, 60% β-glucan, and 1.3% chitin could inhibit the growth of the fungus *Colletotrichum higginsianum* by activating resistance to fungi through the Salicylic Acid (SA)-dependent pathway. β-glucan has been found to have a role in decontaminating toxin that remain in the environment including in water, with the advantages that the toxicity is biologically reduced, saving mammalian cells [21,22]. Meanwhile, β-glucan and its cationic derivatives show decontamination effects by means of their polycation structure, which easily react with the fungi’s biological membrane, and show antifungal activities [22].

The antifungal activity of *P. anomala* is related to cell wall hydrolysis (β-glucanase-induced lysis) and/or the production of volatile metabolites (e.g., ethyl acetate) whereas *S. cerevisiae* produces killer toxic compounds (e.g., K1 peptide toxin) that disrupt plasma membrane integrity [23]. Kusumaningtyas [24] stated that yeasts, such as *S. cerevisiae*, can act as a biocompetitor and can gradually reduce the growth of *A. flavus* through the activity in the production of phenol. Yeasts show antagonistic effects against *A. flavus*, and can thus be used as biocontrol agents or decontaminants to protect food products that are at risk of being attacked by *A. flavus* mold or aflatoxin residues [25].

In addition to antifungal activity, some yeasts and β-glucans from yeast cell walls also show binding activity against toxins, especially those produced by *Aspergillus* sp. *Pichia anomala* produces a volatile compound 2-phenylethanol (2-PE), which is able to inhibit the growth of *Aspergillus flavus* and the production of aflatoxins in pistachio trees [26]. Glucans can not only play a role in mycotoxin absorption but can also directly reduce aflatoxin production. (1,3)-β-D-glucan with side chain (1,6)-β-D-glucan can regulate the presence of aflatoxin [21].

Yeast has shown an ability to reduce aflatoxins through an isotherm adsorption mechanism [27]. β-glucan from yeast cell walls has potential as a mycotoxin binder, with an adsorption mechanism involving different adsorption centers and can easily access hydrogen bonds and ionic or hydrophobic interactions [28]. Glucans can absorb up to 50% of toxin molecules, such as aflatoxins, which most likely occur through hydrogen bonding between the hydroxyl groups, lactones, and ketones of the toxin molecules by single helix and van deer Waals glucan interactions between the phenyl group and part of the β-D-glucopyranose [29,30]. The degradation of aflatoxins (AFB1 and AFB2) involves an open lactone ring and allows decarboxylation to occur at temperatures below 150 °C [31,32].

The yeast cell wall containing β-glucan can absorb up to 29% of aflatoxin B1 (AFB1) depending on the concentration of mycotoxins [28]. The adsorption capacity largely depends on the yeast and mycotoxin composition, but no direct correlation between yeast composition and adsorption capacity has been found, which confirms that the adsorption of mycotoxins by yeasts involves a complex phenomenon [33]. Thus, yeast and β-glucan extracted from yeast cell walls isolated from tofu wastewater could inhibit the growth of *Aspergillus flavus* and also reduce the toxins produced, namely aflatoxins (AFB1 and AFB2). This study aimed to isolate and identify native yeast from tofu wastewater and determine the potential of native yeast and β-glucan extracted from the yeast cell wall as an *A. flavus* growth inhibitor and aflatoxin (AFB1 and AFB2) reducer.

## 2. Materials and Methods

### 2.1. Native Yeast Isolation and Characterization

Fresh tofu wastewater was prepared and 5 mL were collected and placed in a test tube, which already contained 45 mL of 0.85% NaCl. The test tube was shaken using a vortex until the solution was homogeneous. The sample was diluted up to 10^−4^. One milliliter of the diluted sample was taken and placed in a petri dish and then 15–20 mL of YMA medium (47–50 °C) were poured into a petri dish and shaken so that the sample spread evenly. After the media solidified, the petri dish was incubated upside down at ±30 °C for 48 h. Colonies growing on YMA media were then observed. Then, macroscopically different cultures were inoculated on streaked solid agar media. The inoculants were then incubated at 30 °C for 48 h, and then repeated until a uniform culture (pure culture) was obtained [34,35].

The obtained yeast isolate was tested for morphological characteristics and molecular identification to determine the native yeast species. The morphological analysis of yeast was observed under a microscope, while identification of native yeast species was carried out by polymerase chain reaction (PCR). Identification of native yeast from tofu wastewater was carried out molecularly by identifying its genome based on the DNA base sequence of the internal transcribed spacer (ITS) region. The primers used were ITS1 (5’-TCCGTAGGTGAACCTGCGG-3′) as a forward primer and ITS4 (5’-TCCTCCGCTTATTGATATGC-3’) as a reverse primer [36].

The analysis of the sequencing results begins with contigting the DNA base sequences for the ITS region coding using the Contig Assembly Program (CAP) application, which is integrated with the MEGA X software. The contiguous sequences were aligned with the sequence data contained in the Genbank database using the Basic Local Alignment Search Tool (BLAST), which is integrated with NCBI [37]. The phylogenetic tree of native yeast isolates was created using MEGA X software with the neighbor joining (NJ) method, which was tested statistically with the 1000 replicates bootstrap test. The NJ method selects sequences that, when combined, will provide the best estimate of the branch length that most closely reflects the actual distance between the sequences.

To determine the growth pattern of native yeast, the isolate was grown in tofu wastewater. The pH of the pasteurized tofu wastewater used as growth media was then measured at 0, 24, 48, 72, and 96 h. In addition, the growth curve of native yeast isolate was obtained based on the value of the optical density (OD) measured [38].

### 2.2. β-Glucan Extraction from Native Yeast Cell Wall

Extraction was carried out based on Pengkumsri et al. [39]. First, 150 mL of tofu wastewater as isolate growing media were centrifuged at 10,000 rpm at 25 °C for 30 min to separate solids from liquids, and then the supernatant was discarded and the biomass residue was weighed as the weight of cell biomass. The cell biomass was then hydrolyzed with distilled water at pH 5.0 (adjusted to 1.0 M HCl) to break down the cell walls and incubated for 48 h at 50 °C to complete cell wall breakdown. Then, it was reheated at 80 °C for 15 min in a water bath. Then, the sample was centrifuged at 7500 rpm at 25 °C for 10 min.

The autolyzed cells were mixed with 20 mL of 1.0 M NaOH. Then, they were incubated at 80 °C for 2 h with a stirrer. Then, the sample was centrifuged at 7500 rpm for 25 min at 25 °C. The pellets obtained were dissolved in 20 mL of 1.0 M CH_3_COOH and incubated again at 80 °C with a stirrer for 2 h. The pellets were then centrifuged again at 7500 rpm for 25 min at 25 °C. The pellets were taken and then washed with sterile distilled water 3× and freeze dried. The wet β-glucan biomass was freeze dried at −50 °C for 24 h and the result of this drying was weighed as the mass weight of β-glucan produced.

### 2.3. Antifungal Activity Test towards A. flavus

The method of Novrianti et al. [40] used in the antifungal activity test was the well diffusion method of pour plate culture-grown *A. flavus* FRR3961 obtained from Food and Nutrition Culture Collection, Center for Food and Nutrition Studies Universitas Gadjah Mada, Indonesia. Prior to the antifungal test, the initial microorganism was calculated for each native yeast and *A. flavus* using the McFarland 0.5. According to Ernst and Rogers [41], the number of colonies at OD_625_ of 0.5 McFarland is approximately equivalent to 3 × 10^6^ cfu/mL yeast *Candida* and *Aspergillus* conidia. In the diffusion well method, 30 L of native yeast culture and β-glucan were added into the well.

Inhibition of *A. flavus* growth by native yeast isolates and β-glucan extract was carried out through macroscopic observations and described the inhibition [42]. Microscopic observations were done to determine in detail the antifungal activity produced by native yeast isolates and β-glucan extract in inhibiting the growth of *A. flavus*. This microscopic observation was carried out by comparing the growth of *A. flavus* as a control without treatment and inhibition by the antibiotic ketoconazole as an inhibitory positive control. The antifungal activities were identified descriptively based on the microscopic identification results showing the density reduction of the *A. flavus* cell.

### 2.4. Aflatoxin-Reducing Activity Test

Native yeast isolates and β-glucan extract obtained in previous studies were tested for their effectiveness in reducing aflatoxin B1 and aflatoxin B2 by measuring the total aflatoxin levels in the control and samples using HPLC LC-MS/MS [43,44]. The control was as much as 10^6^ CFU/mL of *Aspergillus flavus* grown on PDB media, while the yeast test sample taken from as much as 10^6^ CFU/mL native yeast isolate. The β-glucan test sample was a control with β-glucan dissolved in acetate buffer pH of 5 added [45]. The percentage of toxin-reducing activity was calculated using the following formula:

(1) $$\%\;\mathrm{Toxin}\;\mathrm{reduced}=\frac{Control\;Aflatoxin\;Content-Samples\;Aflatoxin\;Content}{Control\;Aflatoxin\;Content}\;\times \;100\%\;$$

Descriptive statistics were used to analyze the results of the yeast growth, pH change, β-glucan production, and the aflatoxin-reducing activities. The data collected were used to calculate the average and standard deviation and then the results were descriptively analyzed.

## 3. Results

### 3.1. Isolation and Identification of Native Yeast from Tofu Wastewater

The results of the isolation of tofu wastewater native yeast showed that there was one type of isolate with an average colony count of 1.9 ± 0.14 × 10^2^ CFU/mL. The number of yeast colonies isolated was not as much as the number of bacteria.

Based on microscopic observations (Table 1), the characteristics of native yeast cells isolated from tofu wastewater had a length ranging from 2.95–6.20 μm, were oval and round or ovoid shaped, and reproduced by cell budding. The two yeasts were characterized by an ovoid cell shape with cell lengths of 5–6 and 1.2–10.8 μm, respectively.

**Figure 1:**
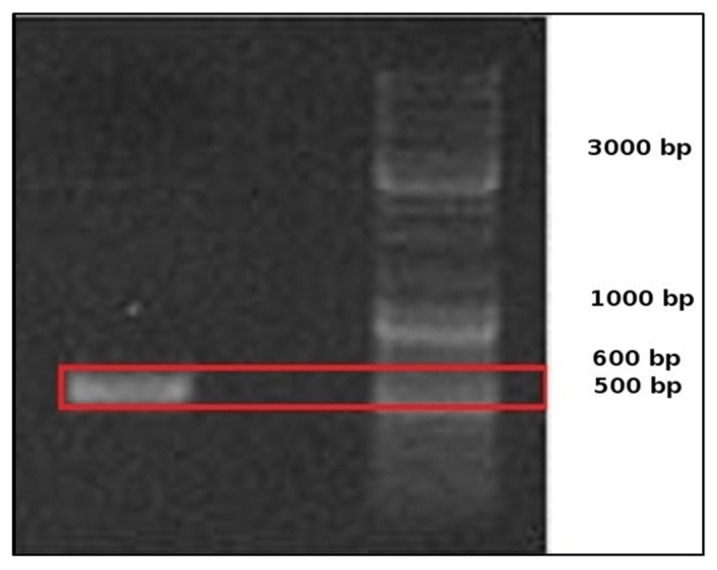
PCR amplification of tofu wastewater native yeast isolate in agarose gel.

The results of PCR amplification of the native yeast isolate DNA extract produced DNA fragments with a band length between 500 and 600 bp, which can be seen in Figure 1. Based on the BLAST results (Table 2), the native yeast isolate was 100% identical to the *Pichia norvegensis* culture sequence CBS: 1953, which was registered with Genbank with the access number KY104639.1. This indicated that the native yeast isolate had an identical chromosome number, genome size, and gene function with *Pichia norvegensis* culture CBS: 1953. Species identification was determined from the percentage identity value obtained through BLAST analysis, and if the homologous DNA content ranged from 60–100%, it was considered an identical species, 20–60% was considered a closely related species, and <20% was considered a different species.

### 3.2. Phylogenetic Tree Reconstruction of Native Yeast Isolated from Tofu Wastewater

**Figure 2:**
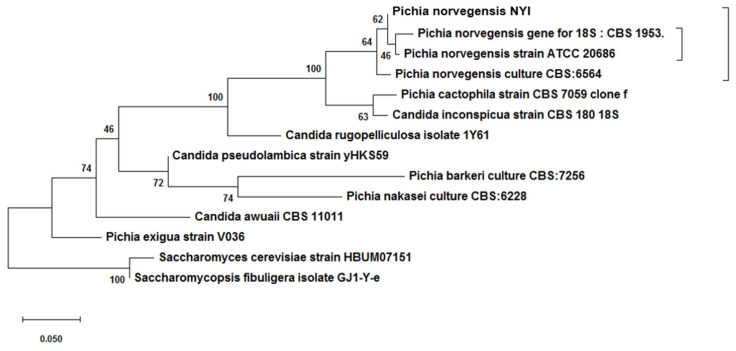
Phylogenetic tree of *Pichia norvegensis* NYI.

Based on the phylogenetic reconstruction results shown in Figure 2, *Pichia norvegensis* NYI is monophyletic with *Pichia norvegensis* gene for 18S: CBS 1953, *Pichia norvegensis* strain ATCC 20686, *Pichia norvegensis* culture CBS: 6564, and closely related to *Pichia cactophila* strain CBS 7059 clone f and *Candida inconspicua* strain CBS 180 18S with a bootstrap value of 100.

### 3.3. Pichia norvegensis NYI Growth

**Figure 3:**
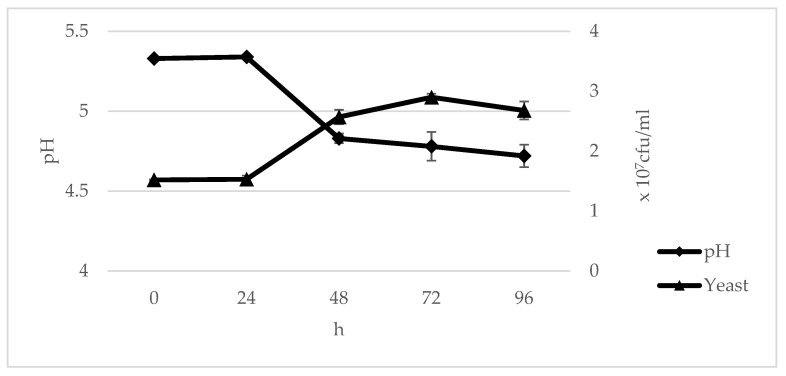
*Pichia norvegensis* NYI growth and changes in the pH of tofu wastewater as growth media.

Based on the results (Figure 3), it can be seen that the pH level of the tofu wastewater decreased day by day while the number of yeast cells increased until 72 h and decreased by 96 h. The tofu wastewater in the early hours was already acidic, as shown by the pH value of 5.33. The results also showed that the yeast growth and pH change at 48–96 h were not significantly different based on the standard deviation.

### 3.4. Production of β-Glucan from Tofu Wastewater by Native Yeast Isolate

**Figure 4:**
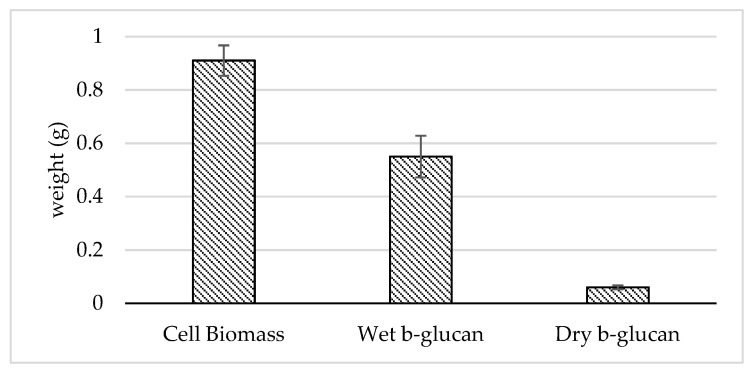
The results of β-glucan production from *Pichia norvegensis* NYI.

Based on the results of the production of β-glucan (Figure 4), *Pichia norvegensis* NYI, with a total biomass weight of 0.91 g, produced 0.55 and 0.06 g of wet and dry β-glucan, respectively, so that the yield of crude β-glucan was 6.59%. From the results, it can also be concluded that the extraction of β-glucan from the cell biomass resulted in a significantly different yield from every step of extraction.

### 3.5. Antifungal Activity of Native Yeast Isolate and β-Glucan

**Figure 5:**
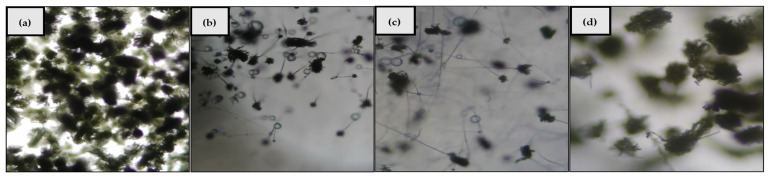
Microscopic observation of antifungal activity of the treatment of (**a**) control (*Aspergillus flavus*); (**b**) positive control (ketoconazole); (**c**) *Pichia norvegensis* NYI; (**d**) β-glucan.

Based on the microscopic observations, it was observed that *A. flavus* only produced sterile spores on inhibition by *Pichia norvegensis* NYI (Figure 5c) while fungal spores were still formed on inhibition by β-glucan (Figure 5d) but in smaller amounts than the control of *Aspergillus flavus* (Figure 5a). The inhibitory activity by *Pichia norvegensis* NYI was seen to be almost the same as that of the positive control of ketoconazole (Figure 5b).

### 3.6. Aflatoxin-Reducing Activity of Native Yeast and β-Glucan

Based on the results shown in Table 3, *Pichia norvegensis* NYI and β-glucan could reduce AFB1 levels, and also decreased AFB2 levels. The highest reduction of AFB1 shown by *P. norvegensis* NYI, and the highest reduction of AFB2 was shown by β-glucan, while total AFB reduction was shown by both treatments. The standard deviation indicated that the treatments of *P. norvegensis* NYI and β-glucan resulted in significantly different AFB1 and AFB2 reduction, while it was not significantly different for total aflatoxin reduction.

## 4. Discussion

### 4.1. Isolation and Identification Native Yeast from Tofu Wastewater

Tofu wastewater in this study is a mixed wastewater from the soybean cooking process, during which there is an increase in temperature as soybeans change into soybean porridge. These changes result in the presence of nutrients in soybeans, which are also wasted in the wastewater. Chua and Liu [46] stated that the wastewater content that can be used as a yeast growth nutrient includes 1% of carbohydrates of mainly stachiose and sucrose, 0.1–0.8% of protein, 0.4–1.0% of fat, and about 0.4% of minerals.

Tofu wastewater is a better medium for bacterial growth culture [47]. Xu et al. [8] stated that the total number of bacteria, yeasts, and molds isolated from tofu wastewater was 7.35 log CFU/mL for bacteria, 4.39 log CFU/mL for yeast, and <2 log CFU/mL for mold. Native yeast isolated from tofu wastewater was similar to yeast isolated in the study of Ebabhi et al. [48] and Khattab et al. [49], namely *Candida tropicalis* and *Pichia caribbica*.

Yeast colonies isolated from 10^−1^ to 10^−4^ dilutions had the same characteristics, namely bone white color and a round shape in YMA media with no hyphae (Table 1). Yeasts are easily distinguished from bacteria because of their larger size and different morphology [50]. Native yeast colonies isolated from tofu wastewater on petri dishes are 0.2–0.3 cm in size, so the colonies are quite clearly visible from a not-too-close viewing distance. This yeast also appears on the surface of the media. Yeasts are also different from molds that can be seen in their appearance whether they have hyphae or not.

Based on Table 1, native yeasts show similar characteristics between *Candida* sp. and *Pichia* sp. According to Ebabhi et al. [48], the isolation of *Pichia caribbica* and *Candida tropicalis* showed creamy white macroscopic characteristics. *Candida tropicalis* and *Pichia kudriavzevii* showed different colony characteristics on solid medium. *Candida tropicalis* was white, smooth, and butyrous, while *Pichia kudriavzevii* had a butyrous appearance, and was bright cream in color [49].

The amplified PCR product was then sequenced to determine the sequence of its nucleotide bases. The results of the sequencing were then investigated by contiguous DNA base sequences encoding the ITS region from the ITS1 and ITS4 sequences. The FASTA contig data were then aligned using the Basic Local Alignment Search Tool (BLAST) on the NCBI website to compare the nucleotide sequences in Genbank and calculate their statistical match so that the yeast species with the highest identification could be identified. The characteristics of sequences that had high identification had the same maximum score and total score, query coverage up to 100%, e-value of 0.0 (zero), and percentage identify of 100% [51].

The query coverage value on *Pichia norvegensis* culture CBS: 1953 showed the highest value, which was 100%, with a maximum score and a total score of 737. Koonin and Galperin [52] stated that the total score shows the total value of base pairs, the maximum score shows the value of the similarity (identical) base pairs, and query coverage shows the percentage of nucleotide samples used in the BLAST analysis. The higher the maximum score, the higher the level of identification. The e-value of 0.0 also indicates that the native yeast isolate sequence has a very high identification with *Pichia norvegensis* culture CBS: 1953. The e-value is an estimated value that provides a statistically significant measure of both sequences. A lower e-value indicates a higher level of homology between the two sequences.

Based on the identification results, the native yeast isolate was a species of *Pichia norvegensis* in accordance with the hypothesis that stated that the identified yeast isolate from tofu wastewater was *Pichia* sp. *Pichia norvegensis* is usually found in dairy products and fermented products, including mozzarella cheese, butter, environmental dairy and yogurt, boza (low-alcohol drink produced by fermenting barley, oats, millet, maize, wheat, or rice), and tempeh [53,54,55,56].

### 4.2. Phylogenetic Tree Reconstruction of Native Yeast Isolated from Tofu Wastewater

The bootstrap test of 100–1000 replicates is effectively used to estimate the confidence level of a phylogenetic tree [57]. The larger the bootstrap replication, the higher the truth level of the reconstructed tree topology based on the distribution of characters in the data, which is strongly influenced by random effects [58]. A bootstrap value above 70 indicates relatively more stable data and the grouping is acceptable [59].

The reconstruction of the phylogenetic tree of native yeast isolates (*Pichia norvegensis* NYI) placed *Saccharomyces cerevisiae* strain HBUM07151 and *Saccharomyces fibuligera* isolate GJ1-Y-e as outgroups (Figure 2). According to Hidayat and Pancoro [60], outgroups are needed and form a tree that is divided into apomorphic and plesiomorphic characters. Apomorphic characters are characters that change and form monophyletic groups in the ingroup, while plesiomorphic characters are primitive characters (ancestors) found in the outgroup. Synapomorphic characters are inherited characters and are found in monophyletic groups.

Ganter and Quarles [61] stated that based on randomly amplified polymorphic DNA (RAPD) analysis, *Pichia norvegensis* and *Pichia cactophila* are species in the same genus and are close to each other, although they have separate lineages. *Pichia norvegensis* is a heterothallic species while *Pichia cactophila* is a homothallic, which can be isolated from opuntia fruit. *Pichia norvegensis* is a teleomorph of *Candida norvegensis* and is closely related to *Candida inconspicua* with a susceptibility pattern similar to that of *Candida krusei*, which is intrinsically resistant to fluconazole [62]. *Pichia cactophila* is a teleomorph of *Candida inconspicua*, which has been identified as molecularly related to sexual reproduction and genome assembly [63].

### 4.3. Pichia norvegensis NYI Growth

At 0 and 24 h, the pH of the media and the number of *Pichia norvegensis* NYI showed a flat curve. This is because the yeast was still in the adaptation phase during this time, so the yeast had not yet experienced significant growth. From 0 to 24 h, the lowest growth rate was shown, which is called the adaptation phase or the lag phase, where the yeast is still adapting to its growth environment [64]. Meanwhile, Deschuyffeleer et al. [65] stated that the *Pichia anomala* yeast lag phase occurs for a maximum of two days at pH 2.8–4. Tofu wastewater usually has a pH between 3.5 and 6.5 due to the addition of coagulant in the tofu-making process [66]. Coagulation in the manufacture of tofu can be achieved by the addition of salt, acid, or coagulant enzymes. Commonly used acid-type coagulants include glucono-δ-lactone, L-ascorbic acid, and other food-grade acids. The coagulant can decrease the pH of soy milk to the isoelectric point of soy protein.

From 24 to 48 h, a rapid increase in growth began to occur until 72 h. This growth is called the exponential growth phase or the logarithmic phase. In this phase, the greatest yeast cell growth occurred at 72 h, which was estimated to be 2.86 ± 0.06 × 10^7^ cfu/mL. This happened because at that time, *Pichia norvegensis* NYI had adapted to the medium environment so that the nutrients from the tofu wastewater could be utilized optimally and the yeast could divide rapidly and constantly. Marrero et al. [67] stated that the exponential growth phase of *Pichia guilliermondii* started at 24 h due to the nutrient media being used quickly and the number of cells increasing. As a result, the final metabolites that can cause changes in the pH accumulate so that the pH decreases while the OD increases.

From 72 to 96 h of growth, there was a decrease in the number of yeast cells, which is called the final stationary phase. Yeast in the logarithmic phase is followed by a stationary phase without growth, which eventually leads to cell death. The yeast produces primary metabolites in the form of organic acids, which result in the pH of the medium being optimum [35]. After this, the number of cells begins to decrease because it enters the death phase. This is because the primary metabolites produced by the yeast are toxic to the yeast itself.

At 48 h, the pH level of the tofu wastewater began to decrease to 4.85 and continued to decrease until 96 h. This is in accordance with the statement of Narendranath and Power [68] that the degree of acidity of the microorganism growth media will decrease day by day. The optimal pH range for yeast growth varies from pH 4 to 6 depending on the temperature, presence of oxygen, and yeast strain. During growth, it is important for the yeast to maintain a constant intracellular pH. The enzymes that function in yeast cells during growth and metabolism work best at their optimal acidic pH due to the acidophilic characteristics of the yeast. Angulo-Montoya et al. [69] stated that the optimal pH of *Candida norvegensis* is 4.17. Yeast growth in high-acidity conditions is considered to be better because acid can neutralize ethanol produced by yeast cells and make it easier for yeast to continue their growth [38].

### 4.4. Production of β-Glucan from Tofu Wastewater by Native Yeast Isolate

*Pichia norvegensis* NYI was grown on tofu wastewater for 72–96 h and its β-glucan content was extracted. This time was chosen because the growth of the yeast *Pichia norvegensis* NYI was in the final stationary phase during which the growth of yeast cells began to enter the death phase. This condition is the optimum condition for producing β-glucan because the number of cells produced has reached the logarithmic phase. According to Utama et al. [35], the greater the number of cells, the greater the amount of β-glucan produced. β-glucan is a component of yeast cell walls, thus β-glucan can still be extracted even though yeast cells have died [70].

Yeast cell biomass is obtained through autolysis of cells or damaged cell walls to produce β-glucan. β-glucan mass and yeast cell mass weight can be used to determine the percentage of β-glucan produced based on the obtained cell mass [35]. β-glucan from *Pichia pastoris* produced a yield of 11.7% with a purity of 85.3% [71]. The percentage of β-glucan to cell mass obtained from *Saccharomyces cerevisiae* was 6–12% [72]. One of the factors that affects the amount of yield produced is the treatment at the autolysis stage and the β-glucan extraction method. Cell autolysis is a key step for isolating polysaccharides and inactivating yeast cells [71]. At this stage, yeast cells are under stress conditions that are influenced by their environment so that endogenous enzymes will be activated to degrade proteins and other macromolecules in the cell.

Extraction of β-glucan in this study was achieved using acid-base extraction with centrifugation treatment. Many and Vizhi [73] stated that different β-glucan extraction methods affect the yield and recovery of β-glucan. The yield of crude β-glucan produced from the acid-base extraction method was higher than the enzymatic method, namely 49.2% for the acid-base method and 17.9% for the enzymatic method. However, the enzymatic method is considered to be more efficient as indicated by the maximum recovery rate of β-glucan when compared to the acid-base method so that it results in a better yield and purity.

### 4.5. Antifungal Activity of Native Yeast Isolate and β-Glucan

Kusumaningtyas [24] stated that yeast has the ability to act as a biocompetitor caused by competition for space and nutrients and the synthesis of antifungal compounds. Yeasts also produce metabolites that can inhibit the formation of *Aspergillus flavus* spores. *Pichia anomala* produces a volatile compound 2-phenylethanol (2-PE), which can inhibit the growth of *Aspergillus flavus* [26]. *Pichia norvegensis* produces a killer toxin that can inhibit the growth of fungi in both low and high pH conditions [61]. This was also conveyed by Hatoum et al. [74], who found that *Pichia norvegensis* has antagonistic characteristics against microorganisms by releasing antimicrobial components, such as antifungal killer toxin or mycocins. Mycocins are extracellular proteins or glycoproteins that interfere with the function of fungal cell membranes that contain receptors for these compounds [23]. Mycocin production occurs among many yeast genera, including *Saccharomyces, Candida, Cryptococcus, Debaromyces, Kluyveromyces, Pichia, Torulopsis, Williopsis,* and *Zygosaccharomyces* [75].

The inhibitory activity of β-glucan indicated that β-glucan inhibited the sporulation process in *A. flavus*. According to Odhiambo et al. [76], *A. flavus* that begins to grow usually forms white mycelia, which then turn green during sporulation. The color of the fungus depends on the type of medium, pH, age of culture, and sporulation. This was also conveyed by Sudini et al. [77], who stated that sporulation in *A. flavus* can be used as an aflatoxigenic indicator.

Yeast can produce metabolites in the form of ethanol compounds and mycocin compounds that can act as antifungals [26,74]. There are several ways to control the growth of *A. flavus* producing aflatoxins in the presence of phenolic compounds and compounds present in yeast cell walls [31]. In addition, the competition for space and nutrients in the media occurred and caused *Pichia norvegensis* NYI to grow and inhibit the growth of *Aspergillus flavus*. Inhibitory activity by β-glucan was seen in the spore, which was larger than the control and there was a transparent mycelium. According to Krijgsheld et al. [78], germination of spores should occur when the environmental conditions favor fungal growth. This is necessary to ensure spores remain in a dormant state because spores can open and experience stress conditions. Furthermore, environmental factors in the presence of fermentable sugars cause stage one of germination. In the second stage of germination, the diameter of the spore is doubled. Klich [79] stated that *A. flavus* when starts to grow, it usually forms white mycelia, which then turns green during sporulation and then mature *A. flavus* transitions to a darker green color.

### 4.6. Aflatoxin-Reducing Activity of Native Yeast and β-Glucan

*Pichia* sp. can reduce the levels of aflatoxin B1, B2, G1, and G2 starting from the first 48 h by producing the volatile compound 2-phenylethanol (2-PE) [26]. Yeast can reduce toxin levels not because of covalent bonds or metabolism, but because of the presence of cell wall components [70]. The dead yeast cells are also known to still be able to bind toxins. The carbohydrate component of the yeast cell wall is a common site for toxin binding, although the toxin may have a different binding site. The decrease in toxin by β-glucan can be caused by the formation of a toxin-glucan molecular complex on the van der Waals bond through adsorption isotherm because this mechanism will not change the molecular structure of β-glucan; however, there will be a change in energy due to the interaction [27,28,30,80].

Table 3 shows that the aflatoxin decrease by β-glucan was greater than that by *Pichia norvegensis* NYI. This is because the β-glucan that was extracted from the yeast cell wall tends to bind more easily to the toxin while the yeast that is added directly must produce metabolites first to reduce the toxin level, although it is also possibly because of adsorption by the yeast cell wall, but the speed of its binding capacity will be different.

According to Nazhand et al. [81], the level of toxicity of AFB2 is smaller than that of AFB1 because the chemical structure of AFB2 does not have a double bond in the bisfuran ring as in the structure of AFB1. The toxicity level of AFB2 is 100 times lower than that of AFB1 due to the presence of a saturated furan ring, which affects the detoxification process [82]. The detoxification process of aflatoxins releases the double bond of the furan ring and modifies the lactone ring. Carvajal-Moreno [31] stated that the change in the molecular structure by hydrogenation of AFB1 to AFB2 is influenced by the presence of oxidative and reductive compounds that bind to aflatoxins. Differences in molecular weight, degree of branching, triple helical conformation, and solubility can affect the binding affinity of β-glucan to each receptor, leading to multiple and variable signal activation pathways [83].

According to Joannis-Cassan et al. [28], β-glucan derived from *Saccharomyces cerevisiae* could reduce AFB1 by up to 29%. Yiannikouris et al. [30] stated that the adsorption range of AFB1 by β-glucan was 22.8–59.7%, whereas according to Aazami et al. [84], it ranged from 6.30–46.34%. Meanwhile, yeast isolates identified as *Kluyveromyces marxianus* and *Pichia kudriavzevii* showed up to 85% of AFB 1 detoxification, thus indicating their potential as an aflatoxin-detoxifying agent [85].

## 5. Conclusions

Tofu wastewater is a source of a potential native yeast that has antifungal and toxin-reducing activity, especially towards *A. flavus* and aflatoxin. *Pichia norvegensis*, as native yeast isolated from tofu wastewater, showed promising antifungal and toxin-reducing activity towards *A. flavus* and aflatoxin. The activities were shown by the yeast and from β-glucan extracted from the yeast cell itself. *Pichia norvegensis* NYI produced β-glucan through cell wall extraction, with a yield of 6.59%. The antifungal activity of *Pichia norvegensis* NYI and β-glucan showed that both could inhibit the growth of *Aspergillus flavus* up to 11.33 ± 4.93 and 7.33 ± 3.51 mm, respectively. *Pichia norvegensis* NYI and its β-glucan were able to reduce the levels of aflatoxins, with a total reduction of 26.85% and 27.30%, respectively.

*P. norvegensis* NYI and its cell wall, represented by β-glucan, can be used as a solution to replace the use of chemical antifungal that produce hazardous residue. The utilization of tofu wastewater as a growth medium also has potential in reducing water pollution resulting from tofu production. Further development can be done to determine the utilization of other food wastes as growth medium and identify the solubility and other biological activities of the resulting β-glucan to improve the application as pharmacy and health supplementation products.

## Figures and Tables

**Table 1:** Characteristics of native yeast isolated from tofu wastewater.

Parameter	Description	Figure
***Macroscopic***		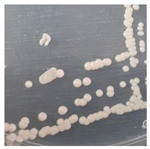
Color	Brownish white	
Colony size	0.2–0.3 cm	
Shape and edge	Round, Smooth	
Elevation	Embossed, Surface	
Appearance	No hyphae, smelly	
***Microscopic***		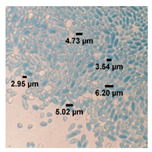
Cell shape	Ovoid	
Cell Size	2.95–6.20 μm	
Reproduction	Budding	
	
	

**Table 2:** BLAST results of native yeast isolate.

Description	Results
Species	*Pichia norvegensis culture* CBS: 1953
Maximum score	737
Total score	737
Query coverage	100%
e-value	0.0
Percent Identity	100%

**Table 3:** Aflatoxin-reducing activity of *Pichia orvegensis* NYI and its β-glucan.

Treatment	Contents (Ppb)			Reduction (%)		
	AFB1	AFB2	Total	AFB1	AFB2	Total
*A. flavus* (control)	5417.69 ± 154.41	173.31 ± 20.93	5591.00 ± 144.34			
*A. flavus + P. norvegensis* NYI	3954.38 ± 53.50	133.24 ± 1.13	4087.62 ± 54.63	36.97 ± 3.07	23.01 ± 3.82	26.85 ± 2.87
*A. flavus +* β-glucan	3946.81 ± 20.93	116.62 ± 0.49	4063.43 ± 21.42	27.13 ± 1.69	32.59 ± 4.20	27.30 ± 1.49
